# Comparison of the SARS-CoV-2 BD Veritor Nasal Antigen Test with Nasopharyngeal Reverse Transcription-PCR in Symptomatic Patients

**DOI:** 10.1128/spectrum.00190-22

**Published:** 2022-08-29

**Authors:** Paula Koga, Maira Maluf, Fabiane Nunes, Juliana Campos, Lívia Gazarini, Tatiane Borghoff, Glaís Libanori, Marinês Martino

**Affiliations:** a Clinical Laboratory Microbiology Department, Hospital Israelita Albert Einstein, São Paulo, São Paulo, Brazil; b Becton and Dickinson Company, BD Life Sciences – Integrated Diagnostic Solutions, São Paulo, São Paulo, Brazil; University of Arizona/Banner Health

**Keywords:** SARS-CoV-2, COVID-19, rapid tests, rapid antigen test, POC test, Veritor test, point-of-care test, coronavirus, RT-PCR, immunochromatographic, point-of-care testing

## Abstract

This study evaluated the BD Veritor system for rapid detection of SARS-CoV-2, an immunochromatographic point-of-care test, by comparing it with a standard reverse transcription-PCR (RT-PCR) methodology using samples from symptomatic patients. Samples from 146 symptomatic and 2 asymptomatic patients between the 1st and the 40th day of infection were evaluated. The nasopharyngeal and/or oropharyngeal swabs were inserted in a tube containing 0.9% saline solution and stored at refrigerator temperature until the moment of use. The samples were first tested with the Xpert Xpress SARS-CoV-2 (GeneXpert) kit (RT-PCR method), and the cycle thresholds (*C_T_*s) for the E and N2 genes encoding the SARS-CoV-2 envelope and nucleoprotein, respectively, were established. Subsequently, the same samples were tested using the Veritor rapid test. We analyzed the *C_T_*s of the N2 gene, which is detected in both methodologies, and observed sensitivities of 100%, 98.8%, 89.6%, and 82.7% for the *C_T_*s of <25, <27, and <30 and all the *C_T_*s, respectively. The greatest sensitivity was observed when we performed the test on patients within 5 days of symptom onset. The BD Veritor system’s workflow is simple and fast, taking approximately 16 min from sample preparation to obtaining the test result. In addition to its satisfactory sensitivity, with results that correlate with those of the RT-PCR, the BD Veritor analyzer instrument reduces the subjectivity of unaided visual readings and consequent potential variation in result interpretation. Therefore, our results showed that the BD Veritor diagnostic test can provide a rapid and accurate diagnosis for SARS-CoV-2.

**IMPORTANCE** This study provides important and useful information, especially for diagnostic laboratories, since the results show that the BD Veritor system can provide a fast and safe point-of-care antigen diagnostic test for rapid detection of COVID-19 that has high sensitivity, reproducibility, and accuracy.

## INTRODUCTION

Coronaviruses are enveloped viruses, with large, positive-sense RNA genomes (1). Coronaviruses cause respiratory infections in a variety of animals, including birds and mammals (2). Seven coronaviruses are recognized as pathogens in humans. Seasonal coronaviruses are generally associated with flu-like syndromes. In the last 20 years, two of them have been responsible for very virulent epidemics of severe acute respiratory syndrome (SARS): the SARS epidemic that emerged in Hong Kong (China) in 2003, with a lethality of approximately 10% (3), and the Middle East respiratory syndrome (MERS) that emerged in Saudi Arabia in 2012 with a lethality of around 30% (4).

SARS-CoV-2, a new coronavirus that produced a respiratory disease and a serious inflammatory response, was identified in December 2019 in Wuhan province, China, and was subsequently given the name Coronavirus Disease 2019 (COVID-19) (5). On 11 March 2020, after the disease reached more than 115 countries, a pandemic was declared by the World Health Organization (WHO). Currently, there are more than 559 million confirmed cases and 6.33 million reported deaths worldwide (6). Up to the beginning of March 2022, there were more than 28,842,160 confirmed cases of COVID-19 in Brazil, with 650,000 deaths (7).

According to the Centers for Disease Control and Prevention (CDC), the gold standard test for the diagnosis of COVID-19 is the detection of viral RNA in samples from the respiratory tract using the reverse transcription-PCR (RT-PCR) technique (8).

However, rapid detection tests enable prevention measures, such as isolation, to be implemented more quickly, helping to reduce the spread of the disease. This has prompted the development of a significant number of point-of-care (POC) antigen tests which can be completed in minutes and require little or no laboratory equipment (9). These POC tests are easy to perform, are low cost, and use a technique based on lateral-flow immunochromatographic assays (LFAs) that detect the nucleocapsid protein from SARS-CoV-2 in nasopharyngeal (NP) specimens within 20 min. This makes POC antigen tests ideal for use with patients both in care settings and in the community (10, 11).

## RESULTS

The study included both female (46.6%) and male (53.4%) patients, from 1 year 10 months to 99 years old. The age of the majority of patients ranged from 18 to 59 years (69.6%). Sixteen patients (10.8%) were children up to 12 years old, and 24 patients (16.2%) were older than 60 years of age.

**FIG 1:**
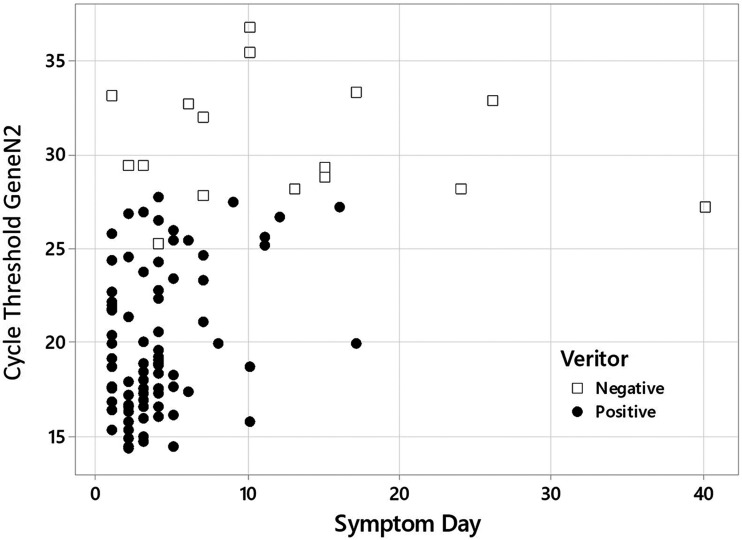
Distribution of *C_T_*s found by RT-PCR for the N2 gene plotted against days from symptom onset of samples that tested positive (black circles) or negative (white squares) in BD Veritor.

Most of the analyzed samples were collected and processed within 5 days of symptom onset and presented cycle threshold (*C_T_*) values for the N2 gene in the RT-PCR below 30 (*C_T_*s of <30) (Fig. 1). The package insert for the BD Veritor system for rapid detection of SARS-CoV-2 recommends the use of the test in samples collected within the first 5 days of the onset of symptoms (11), and indeed, in this study, we observed a high sensitivity rate of 94.7% in samples collected from day 0 to 5 (Table 1). The sensitivity found in this study for samples from patients in the beginning of the syndromic period of COVID-19 (0 to 5 days) was higher than that described in the package insert (84%). Despite the recommendation of the package insert, we analyzed samples collected from patients with more than 5 days of symptoms and observed that the BD Veritor system was still able to detect SARS-CoV-2 in samples from patients up to the 9th day of symptoms with a high sensitivity of 71.4%. BD Veritor also detected the virus in seven samples from patients with 10 days of symptoms or more (Fig. 1).

**TABLE 1:** Sensitivity of the BD Veritor test in samples collected from patients on different days after onset of symptoms that were considered positive in the RT-PCR

Days of symptoms	No. of samples			Sensitivity (%)
	Total	True positive	False negative	
0 to 5	75	71	4	94.7
6–10	15	10	5	66.7
>10	12	5	7	41.7
Overall	102	86	16	84.3

When analyzing the samples from patients with 6 to 10 days of symptoms, we found a sensitivity of 66.7%, and in samples from patients with more than 10 days of symptoms, the sensitivity of the test decreased to 41.7% (Table 1). However, by analyzing all the samples together (overall), the sensitivity obtained was 84.3% (Table 1).

To verify the sensitivity of BD Veritor in respect to samples with different *C_T_*s identified using the GeneXpert RT-PCR platform, we submitted the same samples to the immunochromatographic test. To be considered positive for SARS-CoV-2 in RT-PCR, both the E and N2 genes (which encode the viral envelope and nucleocapsid proteins, respectively) need to be amplified in the samples. Since the nucleocapsid protein is the only target of BD Veritor, in this study we analyzed the *C_T_*s of only the N2 gene (Table 2). For samples with *C_T_* values below 25, 27, and 30 (*C_T_*s of <25, <27, and <30), BD Veritor presented sensitivity of 100%, 98.8%, and 89.6%, respectively (Table 3). When analyzing the samples with all the *C_T_*s together, we found a sensitivity of 82.7% (Table 3).

**TABLE 2:** Sensitivity of the BD Veritor test in samples with different *C_T_*s identified in the GeneXpert RT-PCR platform for the N2 gene, considering all samples in the study

Gene N2	No. of samples			Sensitivity (%)
	Total	True positive	False negative	
*C_T_* < 25	74	74	0	100
*C_T_* < 27	84	83	1	98.8
*C_T_* < 30	96	86	10	89.6
All *C_T_*s	104	86	18	82.7

**TABLE 3:** General validation data comparing the results of samples tested by RT-PCR and BD Veritor

RT-PCR result	No. of samples		
	Total	BD Veritor positive	BD Veritor negative
Positive	104	86	18
Negative	44	0	44

**FIG 2:**
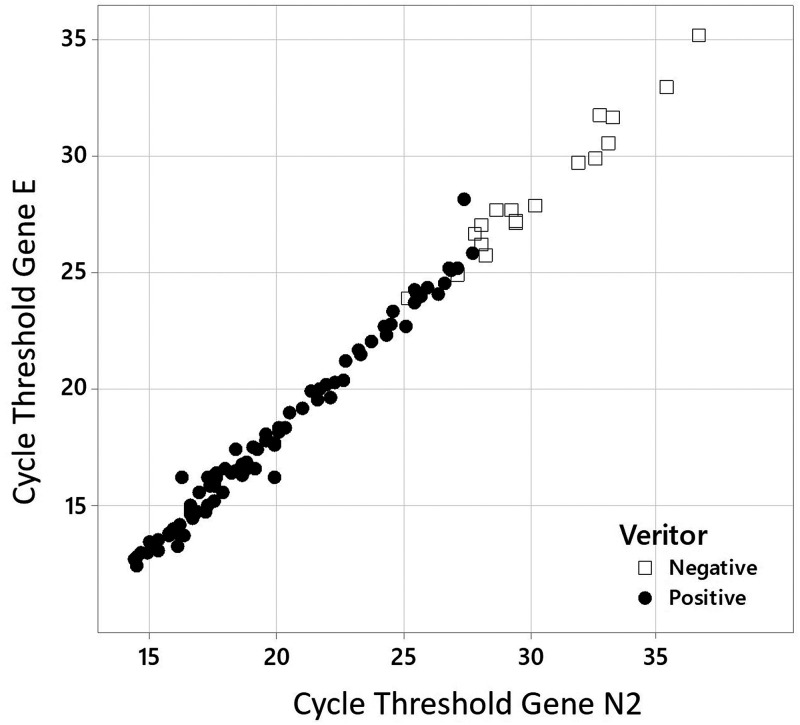
Distribution of *C_T_*s found by RT-PCR for the E and N2 genes in samples that tested positive (black circles) or negative (white squares) in BD Veritor.

As shown in Fig. 2, the highest *C_T_* values of samples diagnosed as positive for SARS-CoV-2 in the GeneXpert platform were 35.10 and 36.70 for the E and N2 genes, respectively. The sample with these highest *C_T_*s tested negative in BD Veritor (Fig. 2). BD Veritor was able to detect as positive samples with *C_T_*s up to 28.06 for the E gene and 27.70 for N2 (Fig. 2). In general, we found good correlation between the positive results obtained by testing the samples with *C_T_*s of <25 for the E gene and *C_T_*s of <27 for the N2 gene identified through RT-PCR and those obtained by BD Veritor (Fig. 2).

**FIG 3:**
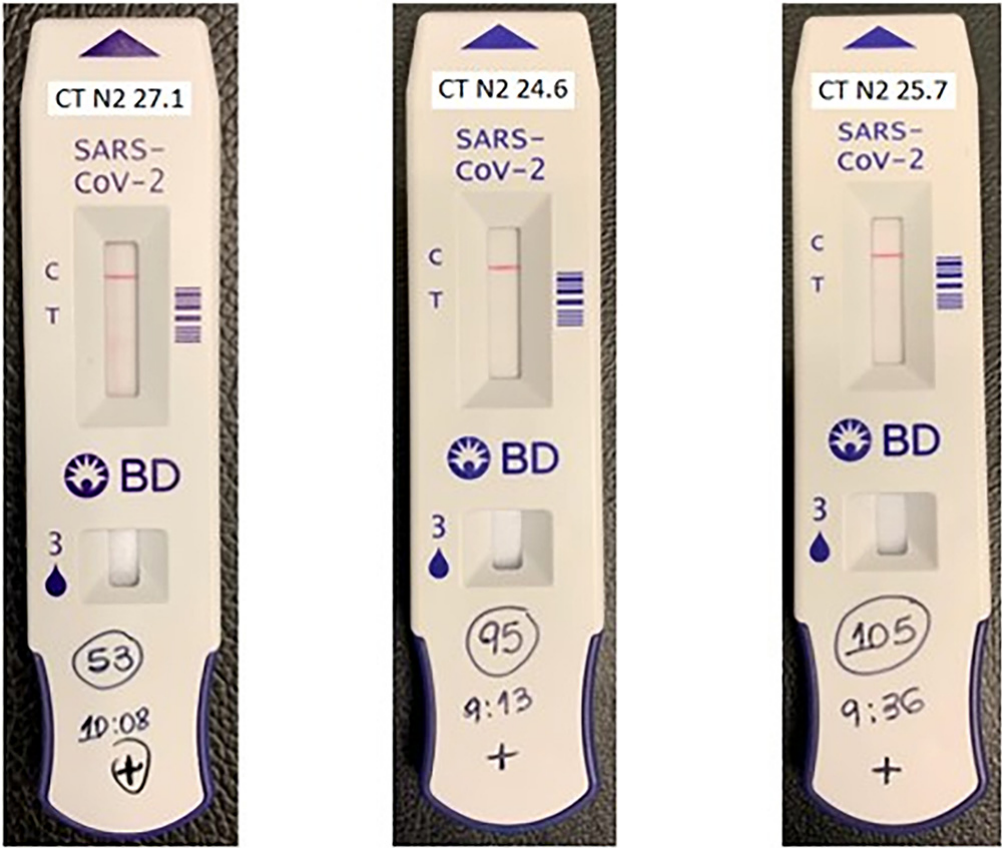
Devices used for testing samples 53, 95, and 105, which were visually negative but had positive results when read by the BD Veritor Plus analyzer, the BD Veritor automated reader.

Samples 53, 95, and 105 were positive when read on the BD Veritor Plus analyzer, as recommended by the manufacturer, even though there was no visual line on the test device. These samples could have been interpreted as negative results, if performed in a kit with visual reading only (Fig. 3). These results show that the automated reading by the BD Veritor Plus analyzer improved the sensitivity of the diagnosis by decreasing the subjectivity of the test reading.

Finally, we analyzed all the samples together that tested negative or positive according to RT-PCR and BD Veritor and observed 100% specificity and 87.8% agreement/accuracy between the two diagnostic techniques (Table 3).

The reproducibility test was performed with two samples previously detected as negative (2033510330 and 2033403215) and two detected as positive (2033510678 and 2033509923). After 3 days of testing, we observed 100% reproducibility between the results of RT-PCR and BD Veritor.

## DISCUSSION

The rapid and correct diagnosis of respiratory viruses is crucial during outbreaks/epidemics/pandemics, such as the SARS-CoV-2 pandemic, to ensure the timely adoption of individual and community measures, the treatment of those with more severe infection, and the isolation of positive patients to reduce the spread of the virus. Although real-time RT-PCR is the gold standard method for SARS-CoV-2 diagnosis, it takes 4 to 6 h to complete sample processing and run the test—from RNA extraction to RT-PCR run—and at least 24 h for the results to be released to the patient (10). This can delay the adoption of prompt infection-control tactics, which is especially important during a public health emergency. Diagnostic tests that can provide faster results can help to overcome this issue (9, 10).

During the SARS-CoV-2 pandemic, several rapid antigen diagnostic tests were quickly developed and marketed worldwide. Most of the rapid antigen tests are lateral-flow immunochromatographic assays that detect SARS-CoV-2 proteins from nasal, nasopharyngeal, and/or oropharyngeal (OP) specimens and give the results within up to 30 min. Despite the time-benefit of the rapid antigen tests, there is a lack of studies directly comparing the results of these tests with the results from RT-PCR tests.

The BD Veritor system kit for rapid detection of SARS-CoV-2 was the first SARS-CoV-2 antigen test to become available to diagnostic laboratories in Brazil during the pandemic. The BD Veritor system consists of an immunochromatographic antigen rapid test which detects the SARS-CoV-2 nucleocapsid protein and can be analyzed in the BD Veritor Plus analyzer, an interpretation instrument that detects positive results unrecognized by the unaided eye. After the addition of the respiratory sample to the immunochromatographic device, the test runs in 15 to 20 min and is read in the analyzer instrument. In this study, we evaluated the performance of BD Veritor compared with RT-PCR as part of the Program of Evaluation of Kits for Coronavirus in Brazil.

The study included 146 samples collected from individuals with different symptom onset times (from 0 to 10 or more days) and 2 asymptomatic individuals who were first tested using the GeneXpert RT-PCR platform and then using BD Veritor. The highest sensitivity of BD Veritor to detect SARS-CoV-2 infection was in symptomatic individuals tested within 5 days of symptom onset (94.7% sensitivity), even when the clinical sample was collected and processed with a different methodology than that recommended in the BD Veritor package insert. BD Veritor is intended to be used to test anterior nasal specimens, but for this study both nasopharyngeal (NP) and oropharyngeal (OP) samples were used, since we aimed to validate the rapid test by using the same collection method used for RT-PCR, so that only a single sample is collected from the patient. Although BD Veritor was able to detect SARS-CoV-2 from the NP and OP samples, additional data are necessary to evaluate the test sensitivity and specificity in NP/OP samples in comparison to anterior nasal specimens. Of note, a recently published literature review reports similar sensitivity and specificity in respect to diagnostic results between different forms of upper respiratory specimens, including anterior nasal and nasopharyngeal swabbing (12).

In general, BD Veritor sensitivity decreased in samples collected further from symptom onset. These results were to be expected, as with symptomatic COVID-19 patients, although viral RNA becomes detectable by RT-PCR in upper respiratory samples as early as the first day of symptoms and continues to be detectable for up to 7 to 10 days, it is more detectable in the first 5 days (13, 14). In samples with *C_T_* values lower than 30, we found sensitivity rates higher than 90%. Moreover, when comparing the positivity rates of samples tested by BD Veritor and RT-PCR, we observed that samples with lower *C_T_* values were more likely to present a positive result in the rapid test.

It is important to highlight a major finding of this study. Three samples diagnosed as positive by RT-PCR, with low *C_T_* values of 27.1, 24.6, and 25.7, were tested in the BD Veritor rapid test device and no visual detectable signal was seen which could be interpreted as a negative result by a human technician; however, when read in the BD Veritor analyzer instrument, the signal was detected and the sample was diagnosed as positive. This result was due to a feature of the instrument designed to detect nonspecific binding and increase the sensitivity of the device to detect positive results that would otherwise be unrecognized by the unaided eye.

In addition to the 100% reproducibility demonstrated, taken together, the results from all the samples analyzed in this study showed that there was a good correlation between the RT-PCR and antigen test positive results, indicating that the BD Veritor system kit for rapid detection of SARS-CoV-2 has satisfactory sensitivity.

A number of points are worth emphasizing, namely, that not all patients included in this study were vaccinated and that during the sample collection period, the Delta variant had as yet not been discovered.

In conclusion, BD Veritor was found to be a simple and fast test, taking approximately 16 min from sample preparation to obtaining the test result, which presented satisfactory sensitivity, with results correlating with the RT-PCR results. Moreover, the BD Veritor analyzer instrument reduces the subjectivity of an unaided visual reading and consequent variation in result interpretation. Therefore, BD Veritor was shown to be a diagnostic test that can provide a rapid and accurate diagnosis for SARS-CoV-2.

## MATERIALS AND METHODS

### Clinical samples.

This study included 148 samples, and the tests were executed in two phases, the first taking place in December 2020 and the second from March to August 2021, with 104 and 44 samples being analyzed, respectively. The tests were performed in different time periods because the supply of tests was limited.

The subjects had a diagnostic RT-PCR test; therefore, nasopharyngeal and/or oropharyngeal swab samples were analyzed in the laboratory at the Albert Einstein Hospital. The hospital has several patient care units spread throughout the city of São Paulo, Brazil, in the neighborhoods of Alphaville, Alto de Pinheiros, Anália Franco, Ibirapuera, Morumbi, and Perdizes. These units provide emergency medical care, and the Morumbi unit also has inpatient beds with wards and intensive care units. The majority of the patients (122 patients) attended the units due to the presence of symptoms, and 26 were hospital inpatients. The tests were performed between 1 and 40 days from the onset of symptoms, with a median of 6 days. The main reported symptoms were runny nose, headache, fever, diarrhea, sore throat, body pain, fatigue, nausea, loss of smell and taste, cough, and dyspnea.

The inclusion criteria for the study were patients with symptoms of COVID-19 who had undergone a RT-PCR test for SARS-CoV-2. There were only 2 asymptomatic patients who had positive results and who were tested only because they were in contact with symptomatic patients who were included in the study. Positive specimens were allocated into three groups depending on the RT-PCR cycle threshold (*C_T_*) result (<25, <27, and <30) for subsequent analysis using BD Veritor in order to verify its sensitivity for SARS-CoV-2 detection in samples with *C_T_*s above 25. The rapid antigen tests in current use in Brazil have, in general, good performance in samples with RT-PCR indicating a *C_T_* below 25. In addition, to verify the specificity of this assay, RT-PCR-negative specimens were also included. For quality control, positive and negative swabs (according to RT-PCR) were tested using the BD Veritor system for rapid detection of SARS-CoV-2.

### BD Veritor system.

The BD Veritor Plus analyzer (Agencia Nacional de Vigilância Sanitária or Brazilian Health Regulatory Agency [ANVISA] registration no. 10033430811, SKU 256066) equipment and the BD Veritor system kit for rapid detection of SARS-CoV-2 (11, 15) (ANVISA registration no. 10033430823, SKU 256082) (Becton, Dickinson and Company) are a new technology in Brazil and Latin America, and the SARS-CoV-2 antigen test was the first launched by BD in diagnostic laboratories in Brazil. The pandemic led to the development of a huge number of antigen tests in Brazil; however, the correlation between the results achieved through these tests and those achieved through RT-PCR was not clear. Therefore, in order to evaluate their performance and standardize and validate the antigen tests, the Program of Evaluation of Kits for Coronavirus was created by the Brazilian Society of Clinical Pathology/Laboratory Medicine together with the Brazilian Society of Clinical Analysis, the Brazilian Association of Diagnostic Medicine, and the Brazilian Chamber of In Vitro Diagnostics and supported by the International Diagnostic Centre of the London School of Hygiene & Tropical Medicine and the Latin American Alliance for the Development of In Vitro Diagnostics. This study is part of this program.

The BD Veritor system is a chromatographic digital immunoassay consisting of an immunochromatographic antigen rapid test that detects the SARS-CoV-2 nucleocapsid proteins in respiratory samples from symptomatic individuals with suspected COVID-19 and uses an interpretation instrument for the qualitative reading of the test result. After sample collection, the swab is placed into an extraction reagent tube, which contains a filter that facilitates the migration of the sample to the test device, especially in the case of samples with excess mucus. The addition of the specimen to the test device is followed by a 15-minute incubation period before being put into the BD Veritor Plus Analyzer. As is usual in immunochromatographic tests, the positive result is determined when the antigen and antibody conjugate and generate a detectable visual signal displayed at the test “T” and at the control “C” position (as a quality control of the device). The BD Veritor interpretation instrument analyzes and corrects nonspecific binding and detects positive results unrecognized by the unaided eye, providing a more reliable diagnosis (11).

The lots of the immunochromatographic kits used in this study to detect SARS-CoV-2 in the BD Veritor instrument (SKU 256082) were as follows: (i) lot 0207621, shelf life 10 December 2020; (ii) lot 0300400, shelf life 4 March 2021; (iii) lot 1052068, shelf life 14 July 2021; and (iv) lot 1120222, shelf life 30 September 2021.

### Sample collection and preparation.

In this study, nasopharyngeal and oropharyngeal specimens were used instead of only nasal specimens as stipulated in the instructions for the use of BD Veritor, since the samples were also used for RT-PCR.

After collection, the nasopharyngeal and oropharyngeal swabs were inserted into a tube containing 0.9% saline solution and stored at refrigerator temperature (2 to 8°C) until the moment of use. The sample was homogenized in a vortex, and 200 μL of saline was transferred to the tube containing the extraction reagent. The test device package was opened, and 3 drops were dispensed into the sample chamber.

The readings of the test devices were performed in the “analyze now” mode, and the results were analyzed later. The parameters of sensitivity, specificity, agreement/accuracy, and reproducibility were evaluated.

### Comparison methodology.

The same samples used above were previously tested with the Xpert Xpress SARS-CoV-2 (GeneXpert) kit, using 300 μL of the sample stored in the 0.9% saline solution, according to the manufacturer’s instructions. The results were reported as negative or positive for COVID-19, and samples with different *C_T_*s were submitted to the BD Veritor test. Samples were considered positive for SARS-CoV-2 if both the E and N2 genes were amplified during RT-PCR.
